# Bibliographical

**Published:** 1895-04

**Authors:** 


### Bibliographical.

We have received recently the fifth edition of Dental Medicine, A Manual of Dental Materia Medica and Therapeutics, by F. J. S Gorgas, A.M., M.D., D.D.S.

This edition has been thoroughly revised and much enlarged. This was necessary, not because the work of the former editions were specially defective, but from the fact that new formulæ are being constantly brought forward, and especially because new therapeutic agents are being introduced in large numbers, some of which, at least, are of value in dental practice, and their description and uses should have a place in such work as this. Among the new remedies added in this edition will be found Sodium-Peroxide, Pental Glycozone, Chloride of Ethyl, Phenosalyl, Boricin, Vaselone, Camphoid, Carbolate of Camphor, Carbolate of Cocaine and many others.

The work has been prepared with reference to both the practitioner and the student as well.

Doubtless there will be some changes and corrections to be made by the author in future editions; but this is about as nearly free from errors and faults as could reasonably be expected.

Dr. Gorgas has made this subject a special study for many years, both in the way of writing and teaching, and we know of no man in this country who is better, if as well, equipped for the preparation of a work on this subject as Dr. Gorgas. He has for years stood in the front rank as a teacher, and the subject of this treatise has been his favorite branch, upon which he has bestowed great attention and labor.

As a work on Dental Medicine it has no superior, if an equal. It is published by P. Blakiston, Son & Co., Philadelphia, and this is enough to say as to the execution of the work. It can be obtained of any bookseller.

